# Inhibition of Ion Channels and Heart Beat in *Drosophila* by Selective COX-2 Inhibitor SC-791

**DOI:** 10.1371/journal.pone.0038759

**Published:** 2012-06-06

**Authors:** Roman V. Frolov, Satpal Singh

**Affiliations:** 1 Department of Pharmacology and Toxicology, State University of New York at Buffalo, Buffalo, New York, United States of America; 2 Department of Physical Sciences, Division of Biophysics, University of Oulu, Oulu, Finland; University of Toronto, Canada

## Abstract

Recent findings suggest that modulation of ion channels might be implicated in some of the clinical effects of coxibs, selective inhibitors of cyclooxygenase-2 (COX-2). Celecoxib and its inactive analog 2,5-dimethyl-celecoxib, but not rofecoxib, can suppress or augment ionic currents and alter functioning of neurons and myocytes. To better understand these unexpected effects, we have recently investigated the mechanism of inhibition of human K_v_2.1 channels by a highly selective COX-2 inhibitor SC-791. In this study we have further explored the SC-791 action on ion channels and heartbeat in *Drosophila*, which lacks cyclooxygenases and thus can serve as a convenient model to study COX-2-independent mechanisms of coxibs. Using intracellular recordings in combination with a pharmacological approach and utilizing available *Drosophila* mutants, we found that SC-791 inhibited voltage-activated K^+^ and L-type Ca^2+^ channels in larval body-wall muscles and reduced heart rate in a concentration-dependent manner. Unlike celecoxib and several other K^+^ channel blockers, SC-791 did not induce arrhythmia. Instead, application of SC-791 resulted in a dramatic slowing of contractions and, at higher concentrations, in progressively weaker contractions with gradual cessation of heartbeat. Isradipine, a selective blocker of L-type Ca^2+^ channels, showed a similar pattern of heart arrest, though no prolongation of contractions was observed. Ryanodine was the only channel modulating compound of those tested additionally that was capable of slowing contractions. Like SC-791, ryanodine reduced heart rate without arrhythmia. However, it could not stop heartbeat completely even at 500 µM, the highest concentration used. The magnitude of heart rate reduction, when SC-791 and ryanodine were applied together, was smaller than expected for independent mechanisms, raising the possibility that SC-791 might be interfering with excitation-contraction coupling in *Drosophila* heart.

## Introduction

Selective inhibitors of cycloxygenase-2 (COX-2) are important non-steroidal anti-inflammatory drugs (NSAIDs), widely prescribed for treatment of arthritis and acute pain. Four coxibs, celecoxib (Celebrex), etoricoxib (Arcoxia), rofecoxib (Vioxx), and valdecoxib (Bextra) were developed and marketed as NSAIDs with reduced gastrointestinal side effects. However, increased risk of cardiovascular side effects, including myocardial infarction, cardiac arrhythmias and stroke, led to eventual withdrawal of rofecoxib and valdecoxib in 2004 and 2005, respectively.

Several studies have demonstrated that celecoxib can interact with various molecular targets, including cellular and enzymatic mechanisms other than cyclooxygenases. For instance, the drug inhibits carbonic anhydrases with nanomolar affinity [1], while at low micromolar concentrations it alters functioning of voltage-activated Na^+^, K^+^ and Ca^2+^ channels [2], [3], [4], [5], induces cytotoxicity towards cardiomyocytes [6], triggers apoptosis and blocks cell cycle progression [1], [7].

We have previously shown that celecoxib can inhibit K^+^ channels in fruit flies and mammals, reducing beating rate and inducing arrhythmia in *Drosophila* heart and cultured rat ventricular cardiomyocytes [8]. Similarly, celecoxib inhibits voltage-activated Na^+^ and K^+^ channels in isolated rat retinal neurons with a strong suppression of spontaneous spiking activity [9]. These effects occur independently from inhibition of cyclooxygenases and involve rapid, direct and reversible action of celecoxib on ion channels (coxibs inhibit COX-2 irreversibly). It has been shown that celecoxib inhibits K_v_2.1 and Shab channels via modification of gating at lower concentrations and channel block at higher concentrations [5], [10]. Also, celecoxib and its inactive analog, 2,5-dimethyl-celecoxib (DMC), but not rofecoxib, can acutely and reversibly up-regulate currents through K_v_7.5 (KCNQ5) cardiac channels in the isolated rat mesenteric artery myocytes and A7r5 rat aortic smooth muscle cells, while simultaneously inhibiting other currents [4]. In addition, celecoxib was shown to inhibit K_v_1.5, K_v_4.3, K_v_7.1, and hERG channels and to alter action potential duration in mouse and guinea pig cardiomyocytes [11], [12].

A surprising variety of coxibs' molecular targets requires further research to better understand possible risks associated with these important drugs. We have recently examined effects of SC-791 on human K_v_2.1channels expressed in HEK-293 cells [13]. The compound, used in the experimental setting [14], [15], [16], [17], has been selected for two reasons, an extremely high selectivity for COX-2 (SC-791 inhibits hCOX-2 with an IC_50_ of 4 nM and hCOX-1 with an IC_50_ of 114 μM [18], a selectivity ratio of 28,500), and its structural similarity to celecoxib [13]. The drug inhibits K_v_2.1 via gating modification, but, unlike celecoxib, it does not induce channel block. In this study we have examined effects of SC-791 on ion channels and heartbeat in *Drosophila melanogaster.* The fruit fly, a widely used model organism, apparently lacks cyclooxygenases [8] and thus is very well suited for basic research on the COX-2-independent action of coxibs. Here we show that SC-791 inhibited voltage-activated K^+^ and L-type Ca^2+^ channels and reduced heart rate in concentration-dependent manner. Unlike celecoxib and several other K^+^ channel blockers, SC-791 did not induce arrhythmia. Instead, application of SC-791 resulted in a dramatic slowing of contractions and, at higher concentrations, in progressively weaker contractions with a gradual cessation of heartbeat.

## Results

### Effects of SC-791 on ionic currents in larval muscles

**Figure 1:**
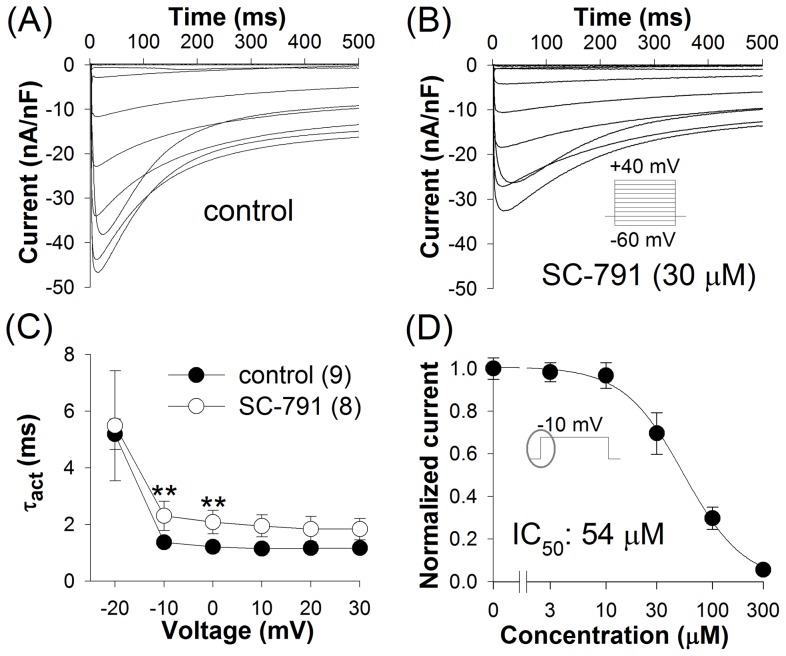
Inhibition of L-type Ca^2+^ channels by SC-791. L-type currents in larval body-wall muscles in control (*A*) and in the presence of 30 μM SC-791 (*B*) were elicited by 500 ms voltage pulses between −60 and +40 mV in 10 mV increments from a holding potential (HP) of −40 mV; averaged traces are shown. (*C*) SC-791 slowed activation of L-type Ca^2+^ channels. Activation time constants were determined by fitting the rising part of the current with a single exponential function; (n), number of experiments. (*D*) Dose-response relation for inhibition of the peak L-type current by SC-791 at −10 mV; the curves in this and following dose-response relations represent fits to Hill equation.

Several ionic currents have been previously identified in *Drosophila* larval body-wall muscles: T-type and L-type Ca^2+^ currents [19], [20], [21], two calcium-activated K^+^ currents, and three voltage-activated K^+^ currents [22], [23]. The latter include Shaker channels, conducting fast activating and inactivating I_A_; Shab channels, mediating relatively slow delayed rectifier I_KS_; and rapidly activating I_KF_ with unknown molecular basis [24]. In this study we have assessed effects of SC-791 on four major currents: L-type current and three K^+^ currents, I_A_, I_KS_, and I_KF_ (Fig. 1*A*, 2*A*). These currents can be separated by electrophysiological, pharmacological and genetic means. The L-type current can be isolated by recording in the presence of K^+^ channel blockers (TEA, 4-AP and quinidine) from a holding potential (HP) of −40 mV (to inactivate T-type Ca^2+^ channels) with Ba^2+^ ions as charge carrier (to prevent muscle contractions and to additionally block K^+^ channels) [20], [24]. The I_KF_ can be recorded from the *Shab* strain in the presence of 4-AP to block the I_A_; the I_KF_ can be completely abolished by 200 μM Cd^2+^ in the external solution [8], allowing recording of I_KS_ from the wild-type larvae. The I_A_ can be recorded from wild-type animals with Cd^2+^ (to inhibit I_KF_) and 100 μM quinidine (to selectively inhibit the I_KS_) in the bath or from the *Shab* in the presence of Cd^2+^ only.

**Figure 2:**
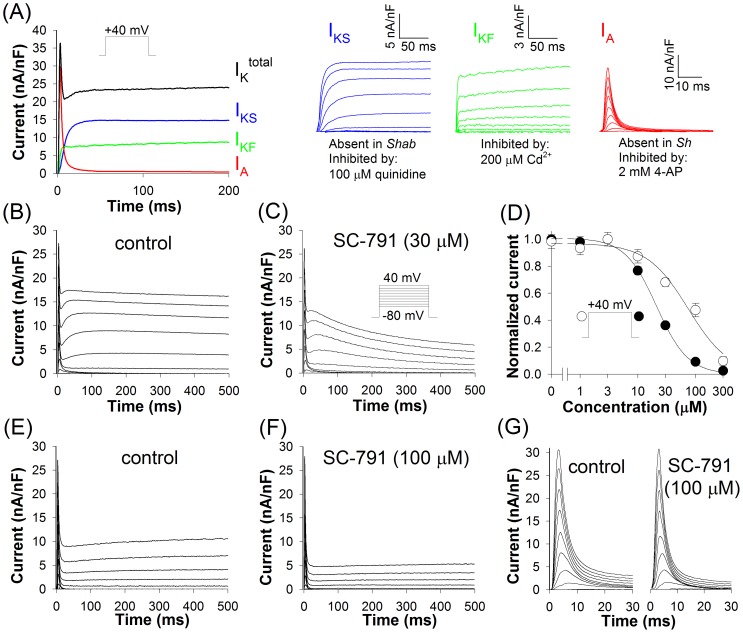
Inhibition of K^+^ currents by SC-791. (*A*) Schematic representation of three voltage-activated K^+^ currents. The average I_KS_ in control (*B*) and in the presence of 30 μM SC-791 (*C*) in the wild-type larvae; currents were evoked by 500 ms voltage pulses between −40 and +40 mV from a HP of −80 mV in 10 mV increments in the presence of 200 μM CdCl_2_ in bath solution to block the I_KF_; the I_A_ can be seen in panels *B, C, E, F* in the beginning of each trace; it was not removed as it did not obscure the effects of SC-791 on other currents. (*D*) Dose-response relations for inhibition of the I_KS_ by SC-791 at 50 ms after the onset (open circles) and at the end of a voltage pulse to +40 mV (closed circles); the number of experiments varied from 5 to 12. The average I_KF_ in control (*E*) and in the presence of 100 μM SC-791(*F*) in *Shab* mutant larvae; the currents were elicited by 500 ms voltage pulses between −40 and +40 mV from a HP of −80 mV in 10 mV increments; the number of experiments for each condition was 8. (*G*) SC-791 did not affect the I_A_; the currents were recorded from *Shab* larvae as in the panels (*B–C*) in the presence of 200 μM CdCl_2_ to block the I_KF_; the number of experiments for each condition was 9.

SC-791 inhibited L-type Ca^2+^ channels in a concentration-dependent manner with an IC_50_ of 54 μM for the peak currents (Fig. 1). The L-type current in the presence of SC-791 was characterized by slower kinetics of activation and inactivation, suggesting modification of channel gating or closed-channel block, the latter can be associated with slowing of activation and inactivation kinetics due to partial unblock of blocked channels at positive voltages.

Figure 2*A-C* demonstrates that the main effect of SC-791 on the I_KS_ was acceleration of inactivation, though the reduction in the peak I_KS_ indicates that the drug might also alter the activation kinetics, similarly to its action on the human K_v_2.1 [13]. The effect of SC-791 on I_KS_ was measured at 50 ms after the onset of a voltage pulse to +40 mV and at the end of 500 ms pulses. The IC_50_s for inhibition of I_KS_ for these two time points were 74 and 21 µM, respectively (Fig. 2*D*). The effect of SC-791 on I_KF_ was relatively small with a noticeable inhibition observed only at 100 μM and higher concentrations (Fig. 2*E–F*); 100 μM SC-791 failed to alter the I_A_ altogether (Fig. 2*G*).

### Effects of SC-791 on heartbeat

**Figure 3:**
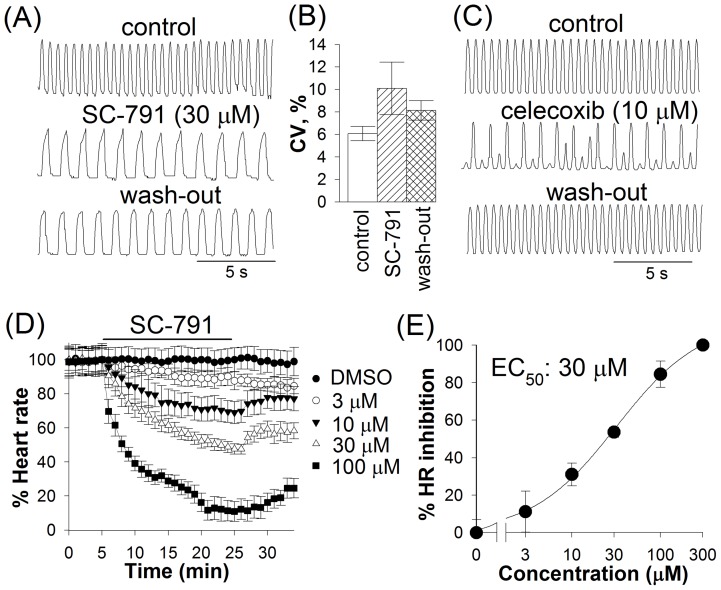
Effects of SC-791 on heartbeat. Traces represent the relative displacement of an edge of the larval heart in the experiments with SC-791 (*A*) or celecoxib (*C*); heartbeat was video recorded immediately before drug application, at 18–20 min after wash-in of 30 µM SC-791 or at 8–10 min after application of 10 μM celecoxib, and at 8–10 min after washing the drug out; (*B*) depicts heartbeat irregularity in the presence of 30 μM SC-791; the coefficient of variation (CV) was calculated as described previously [8]; the number of experiments was 8. (*D*) Average heart rate is shown for 6–10 larvae before drug application, after rapid wash-in of 3 μM, 10 μM, 30 μM, or 100 μM SC-791 (horizontal bar), and after the wash-out; treatment with 0.5% Me_2_SO, the maximum concentration of Me_2_SO used with SC-791, did not change the heart rate significantly. (*E*) A dose-response curve for the effect of SC-791 on heart rate is shown.

The larval heart has a pacemaker region and a non-pacemaking myocardium [25]. Electrical activity in the heart is driven by myogenic pacemaker located in the caudal-most segment of the heart [26]. Larval heart tube myocytes do not express sodium channels; instead, depolarization is provided by L-type Ca^2+^ channels as in the body-wall muscles [27], though L-type Ca^2+^ channels are apparently not involved in the pacemaker activity [27]. Among potassium channels, involvement of Shab, KCNQ and eag in *Drosophila* heartbeat has been documented previously [8], [27], [28], [29]. SC-791 inhibited heartbeat in dose-dependent manner with an EC_50_ (the effective concentration to alter a functional parameter by 50%) of 30 μM (see example in Fig. 3*A*); drug effects developed slowly and usually reached a relatively steady-state level around 20 min after a rapid wash-in (Fig. 3*D*). They included a reduced heart rate and heart wall displacement amplitude and prolonged contraction duration. During contraction, the maximal displacement of heart wall was observed at 160±10 ms after initiation of contraction in control and at 254±17 ms in the presence of 30 μM SC-791. The character of SC-791 effect on heartbeat was distinct from that of celecoxib. In the presence of celecoxib, irregularity of heartbeat with a significant variation in both the peak-to-peak intervals and the amplitude of heart wall displacement was observed, especially at concentrations above 3 μM [8] (Fig. 3*C*). In contrast, analysis of variation in peak-to-peak intervals and in amplitude of heart wall displacement indicated that SC-791 did not increase heartbeat irregularity significantly (Fig. 3*B*). In addition, the effects of SC-791 on heartbeat were largely irreversible.

**Figure 4:**
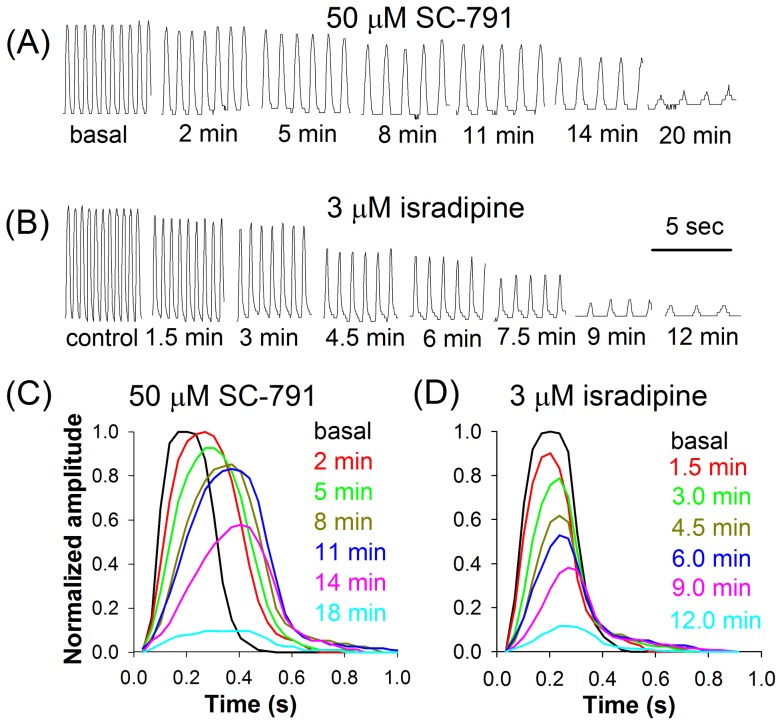
Heartbeat disruption by SC-791 and isradipine. Development of heartbeat stoppage in the presence of 50 μM SC-791 (*A*) or 3 μM isradipine (*B*). The corresponding averaged contraction waveforms demonstrate similarities (decrease in amplitude) and differences (prolongation of contraction) between the effects of SC-791 (*C*) and isradipine (*D*); each waveform is an average of 10 to 20 individual contraction waveforms.

At 50 μM and higher concentrations, SC-791 progressively reduced heart rate, increased duration of heart contractions and decreased contraction amplitude up to the point when heartbeat could no longer be detected: at 100 μM SC-791 heartbeat ceased after 20 min exposure in 3 out of 6 cases, and at 300 μM heartbeat ceased in 3 out of 3 cases after 10 min exposure. Figure 4*A* demonstrates an example of a developing failure of heartbeat at 50 μM SC-791; note that the drug mainly increased the duration of the initial phase of contraction. Once again, this pattern was different from the effects of higher concentrations of celecoxib, which usually involved a sudden halt of beating, often against the background of severe arrhythmia [8].

In a previous study we have shown that effects of celecoxib on heart rate in *Drosophila* primarily result from inhibition of Shab channels [8]. Inhibition of other channels, including L-type Ca^2+^ channels, by low micromolar concentrations of celecoxib has been negligible. In an attempt to understand what could account for the strikingly different character of SC-791 action, we tested if it can be reproduced through inhibition of several ion channels known for their role in *Drosophila* heart. Because SC-791 inhibited L-type Ca^2+^ channels with an IC_50_ of 54 μM, we first tested effects of isradipine on heartbeat. 10 μM isradipine, a selective dihydropyridine blocker of L-type Ca^2+^ channels [19], blocked L-type Ca^2+^ channels in larval body-wall myocytes nearly completely without any effect on K^+^ currents. At higher concentrations, isradipine was able to block K^+^ current with an IC_50_ around 30 μM for the currents at the end of a 500 ms pulse to +40 mV and with a much smaller effect on the peak currents, indicating open-channel block (data not shown). Effects of exposure of heart preparation to 1 or 3 μM isradipine strongly resembled the effects of SC-791: a gradual decrease in heart rate with decreasing amplitude of contractions (Fig. 4*B*). However, in contrast to SC-791, isradipine did not prolong contractions (Fig. 4*C*–*D*). Several other K^+^ channel blockers and toxins were tested, including TEA (indiscriminate K^+^ channel blocker; at submillimolar concentrations it can block K_v_3), quinidine (blocks K_v_2 and other channels), 4-AP (K_v_1), heteropodatoxin (K_v_4), linopirdine (K_v_7), E-4031 (erg or K_v_11), paxilline (K_Ca_1), and apamin (K_Ca_2). Some of these compounds (TEA, quinidine [8], and linopirdine) altered heart rate and induced arrhythmia but none were able to slow contractions (data not shown).

### SC-791 and Ca^2+^ release from intracellular stores

Prolongation of contractions in the presence of SC-791 could not be explained by a reduced Ca^2+^ influx through partially inhibited L-type Ca^2+^ channels (isradipine blocked L-type channels but did not prolong contractions), nor by slowing of L-type current activation (the activation time constants at the voltages above −20 mV did not exceed 3 ms (Fig. 1*C*), i.e. they were two orders of magnitude smaller than the duration of the actual contraction (160 ms in control); it should also be noted that Ca^2+^ channel recordings but not heartbeat measurements were performed using Ba^2+^ as a charge carrier, which slows channel kinetics substantially. On the other hand, it is known that changes in intracellular Ca^2+^ dynamics can alter the velocity of muscle contractions. For example, disruption of ryanodine receptor (RyR)-mediated Ca^2+^ release in RyR loss-of-function mutant flies dramatically slows the muscle contraction velocity and reduces heart rate to 25% of control [30]. Similarly, we found that thapsigargin, inhibitor of sarcoplasmic reticulum Ca^2+^-ATPase (SERCA), which is responsible for replenishment of Ca^2+^ stores in the sarcoplasmic reticulum, can dramatically reduce heart rate and prolong contractions (unpublished observations); this observation is consistent with the previously published report on the crucial role of SERCA in *Drosophila* heartbeat [31]. Moreover, low micromolar concentrations of Cd^2+^ were also able to alter heartbeat drastically, transforming individual contractions into prolonged spastic events with contracted heart muscle unable to relax for many seconds (unpublished observations). We therefore hypothesized that slowing of contractions in the presence of SC-791 could result from modulation of Ca^2+^ release from sarcoplasmic reticulum. As previous studies have described interaction of celecoxib with numerous ion channels and other targets, and this study showed that SC-791 is similar to celecoxib in this regard, and can modulate several dissimilar ion channels, it appeared possible that SC-791 could interact with Ca^2+^ release channels, specifically with the RyR Ca^2+^ release channel, a major component of excitation-contraction (EC) coupling in myocytes, which mediates Ca^2+^ release in response to activation of dihydropyridine (DHP) receptors L-type Ca^2+^ channels. We tested this hypothesis by examining effects of ryanodine on heartbeat separately and in combination with SC-791.

**Figure 5:**
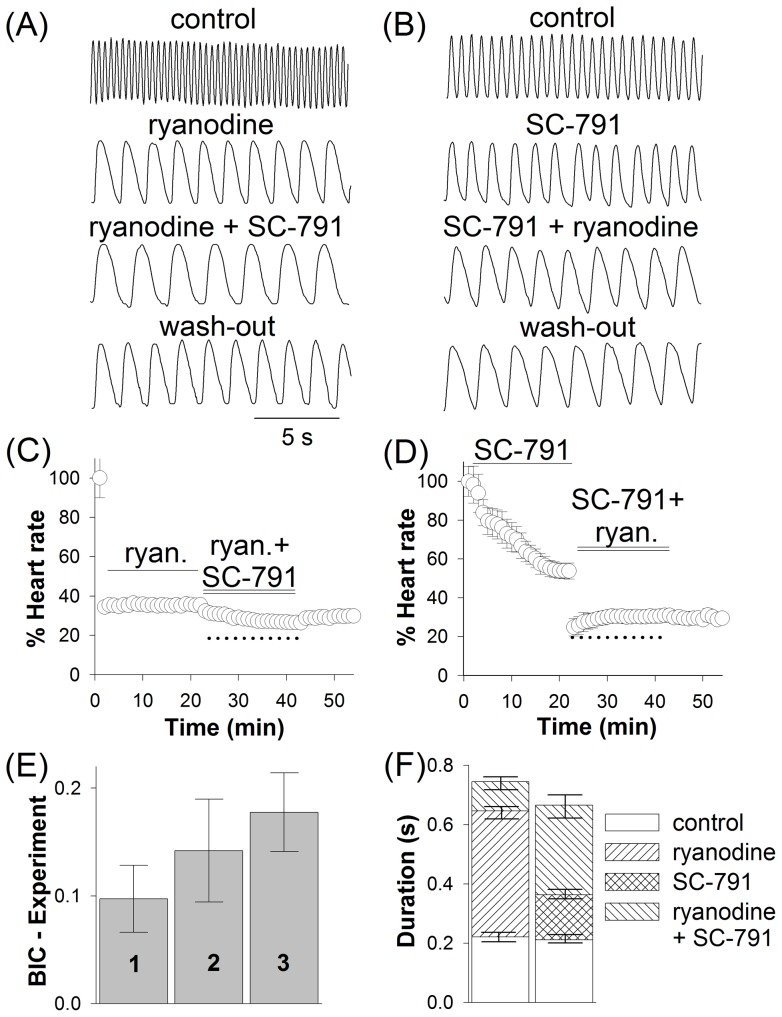
Effects of ryanodine on heartbeat. Typical examples of heartbeat in control, after 20 min application of either 100 µM ryanodine (*A*) or 30 µM SC-791 (*B*) alone followed by 20 min exposure to a solution containing both 100 µM ryanodine and 30 µM SC-791, and after the wash-out. (*C, D*) Time course of the average heart rate in the experiments described in the panels (*A*) and (*B*), respectively; dotted lines indicate the value of BIC (82% heart rate inhibition) (see Results); panels (*C*) and (*D*), averages of 7 and 6 experiments, respectively. (*E*) Bar plot illustrates differences between the BIC of 0.82 and the experimentally determined total fractional reduction in heart rate for three experimental conditions: when SC-791/ryanodine was applied after (1) exposure to ryanodine alone, (2) exposure to SC-791 alone, and (3) without pre-exposure of the preparation to any compound. (*F*) Bar plot summarizes effects of ryanodine and SC-791 on the duration of heart contraction when the chemicals were applied in different order as in the panels (*A*) and (*B*).

Ryanodine is a highly selective modulator of the RyR Ca^2+^ release channels. At nanomolar concentrations ryanodine promotes opening of the RyR, at low micromolar concentrations it locks the RyR in the half-open state, and at concentrations around 100 μM it completely and irreversibly inhibits the RyR [30]. In our experiments, 100 μM ryanodine instantly and irreversibly reduced heart rate with a remarkable prolongation of contractions (Fig. 5*A*). Interestingly, 10, 100, and 500 µM ryanodine reduced heartbeat to a similar degree, by 61±4, 62±2, and 63±5%, respectively, suggesting that all these concentrations are in the saturation range. As even 500 μM ryanodine, the highest concentration used, could not stop heartbeat, these results also indicated that RyR-mediated Ca^2+^ release is an important but not the essential aspect of EC coupling in the larval heart. This conclusion is consistent with the previously published observations [30].

If SC-791 and ryanodine interacted with the same molecular target, i.e. inhibited the RyR, then the total effect of two compounds applied together would be smaller than expected from the Bliss independence criterion (BIC) E^BI^_AB_. The BIC is derived from probability theory as a measure of total effect of two pharmacological agents acting via independent mechanisms. The equation for the zero-interaction effect is E^BI^_AB_ = E_A_+E_B_−E_A_*E_B_, where E_A_ and E_B_ are the fractional single-agent effects of compounds A and B at given concentration [32], [33]. If the experimentally determined effect of a combination of two drugs is smaller than the BIC, then antagonistic interaction between the compounds may be at play; if it is higher, synergism is likely [34]. Based on our experimental results, we calculated the BIC for the combined effects of 100 µM ryanodine (62% heart rate decrease) and 30 µM SC-791 (52% heart rate decrease) and compared it to the experimentally measured effect of the combination of the drugs (Fig. 5). The value of BIC was 0.82, i.e. if the drugs suppressed heartbeat via independent mechanisms, 82% heart rate reduction would be expected in the presence of 100 µM ryanodine and 30 µM SC-791. When solution containing 30 µM SC-791 and 100 µM ryanodine was applied after 20 min exposure to 100 µM ryanodine (Fig. 5*A, C*), the combined effect at the end of 40 min long exposure was the decrease in heart rate by 72±3%. However, when the two drugs were applied after 20 min exposure of heart preparation to 30 µM SC-791, the total reduction in heart rate was 68±5% (Fig. 5*B, D*). Specifically, 30 µM SC-791 reduced heart rate by 52±2% when used as a single-agent (Fig. 5*D*) *versus* 25±5% when SC-791 was applied after exposure to 100 µM ryanodine (P<0.001, ANOVA, Fig. 5*C*). The respective values for ryanodine were 62±3% (Fig. 5*C*) and 48±4% (P<0.02, Fig. 5*D*). Interestingly, when 100 µM ryanodine and 30 µM SC-791 were applied simultaneously without pre-exposure of heart to any drug, the heart rate was inhibited by only 64±4%. Figure 5*E* shows average values of difference between the BIC of 0.82 and the experimentally determined reduction in heart rate in the presence of the two drugs (for three different orders of drug application As the reduction in heart rate was less than the BIC value in all these cases, our results suggested that SC-791 directly or indirectly interfered with the RyR-mediated Ca^2+^ release.

However, if SC-791's influence on the contraction was mediated *only* via the RyR, then no additional effects of the SC-791 on heartbeat would be observed in the presence of 100 μM ryanodine, a saturating concentration almost completely inhibiting the receptor. Yet two such effects were observed: a further reduction in heart rate and slowing of contractions (Fig. 5). While the former could be associated with the consequences of inhibition of L-type Ca^2+^ channels as illustrated in the action of isradipine, the latter could not. Figure 5*F* shows that addition of SC-791 to ryanodine-supplemented bath solution had further decelerated heart wall displacement: from 210 ms to reach the maximal displacement in control, to 645 ms in the presence of ryanodine, and then to 745 ms after adding in 30 μM SC-791.

Two other substances, thapsigargin and Cd^2+^, could disrupt heartbeat and slow down contractions, however, it was not possible to investigate pharmacological independence of their and SC-791's effects due to rapid and irreversible deterioration of heartbeat after their application.

## Discussion

Because of the significance of selective COX-2 inhibitors in contemporary therapeutics, it is important to investigate their action on ion channels and the resulting functional changes at the cellular and organ levels. Recent research demonstrates that low micromolar concentrations of celecoxib and its analogs can modify functioning of a broad range of ion channels via gating modification or channel block, and alter neuronal and myocytes functioning [2], [3], [4], [9]. Because different ion channels can be modulated by coxibs via dissimilar mechanisms with different outcomes (reduction or augmentation of a current) and because specialized cells express specific compositions of ion channels, the end effects of the drugs on cell performance are likely to differ even between cells executing the same function. Indeed, Macias and coauthors have shown that celecoxib can prolong action potential duration in mouse cardiac myocytes while shortening it in guinea pig cardiac myocytes [13]. An additional layer of complexity to this picture is added by possible interaction of coxibs with other molecular targets that could alter cell functioning but are not related to ion channels, such as the coxibs' main receptor, COX-2, or carbonic anhydrases [1], [7]. Thus, although apparently no single ‘ideal’ model organism for studying ion channel-related effects of coxibs exists, the well-studied *Drosophila*, which conveniently lacks cyclooxygenases, provides a good experimental setting for basic research in this direction.

This study demonstrates that even highly structurally similar coxibs, celecoxib and SC-791, can dramatically differ in their effects on *Drosophila* heartbeat. Evidence suggests that these differences originate from dissimilar profiles of ion channel inhibition. Celecoxib inhibits Shab channels in body-wall muscles with an IC_50_ of 10 μM for the currents at the end of a 500 ms pulse to +40 mV (an IC_50_ of 37 μM for the current at 50 ms after the start of the voltage pulse) [8]. At higher concentrations, celecoxib inhibited L-type Ca^2+^ channels (an IC_50_ of 74 μM for the peak current, unpublished observations) and also the I_KF_ (about 50% inhibition at 100 μM) [8]. Accordingly, the drug has reduced larval heart rate with an EC_50_ of 11 μM; its effects on heartbeat are very similar to those observed in *Shab* flies, with a decrease in heart rate, strong arrhythmia and an abrupt ceasing of beat observed at higher concentrations. In contrast, SC-791 inhibited Shab current with IC_50_s of 21 μM (the end of 500 ms pulses) and 74 μM (at 50 ms), and L-type current with an IC_50_ of 54 μM. The heart rate was reduced with an EC_50_ of 30 μM, but in a different manner: with a much slower development of the effect, without substantial arrhythmia, virtually irreversibly, and with prolongation of contractions.

Thus, at lower concentrations (<50 μM) celecoxib mainly affects the I_KS_, whereas SC-791 – the L-type current and the I_KS_. This difference can explain the absence of arrhythmia in the presence of SC-791: celecoxib and SC-791 perturb the balance of ionic currents during action potential in dissimilar ways. By inhibiting K^+^ channels with a higher potency than Ca^2+^ channels, celecoxib might induce arrhythmia by compromising repolarization in the pacemaker cells without measurably affecting depolarization. SC-791, on the other hand, inhibited both Shab and L-type Ca^2+^ channels with a similar potency, which could result in less misbalanced depolarizing and repolarizing currents and, therefore, trigger no arrhythmia (although certain distortions in the action potential waveform would inevitably arise even in such case – from increase in the membrane time constant due to inhibition of conductances, and from non-uniform voltage-dependencies of ion channel inhibition). Such scenario could also explain the differences in the character of heart pauses and stops: blocking repolarization, celecoxib often stops heartbeat abruptly (with a similarly sudden resumption of beating), whereas SC-791 stopped heartbeat after a gradual decrease in the beating rate and contraction amplitude, and without pauses.

Regarding possibly different effects of celecoxib and SC-791 on pacemaker activity, two aspects should be emphasized. First, the ion channels regulating the pacemaker activity in the larval heart appear to be not the same as the ion channels providing for depolarization and signal propagation in the myocardium [27], [35]. One of the key differences is that the L-type Ca^2+^ channels are not important for the function of the pacemaker, while OPQ-type Ca^2+^ channels appear to be critical [27]. It is not known if celecoxib and SC-791 can modulate the latter channels in *Drosophila*, but celecoxib blocked similar channels in rat PC12 cells with lower affinity than the L-type Ca^2+^ channels [2], suggesting that celecoxib might alter the balance between depolarizing and repolarizing currents even more drastically in the pacemaker cells than in the myocardium. Secondly, although heart rate is regulated by the pacemaker cells presumably lacking the L-type Ca^2+^ channels, application of relatively low concentrations of isradipine (Fig. 4*B, D*) was nevertheless capable of reducing heart rate, indicating that either some other pacemaker's ion channels are affected by the drug or myocardium itself is involved in heart rate regulation.

Another explanation for the lack of arrhythmia in the presence of SC-791 could be that as the body-wall muscle and heart channellomes are not the same, the drugs could differentially inhibit other ion channels expressed in heart but not in body-wall muscles. Two such channels associated with arrhythmic heartbeat are KCNQ and erg. Experiments in mammalian experimental systems have demonstrated that celecoxib can inhibit these and many other channels [2], [3], [4], [5]. For instance, application of linopirdine, a highly selective KCNQ inhibitor, reduced *Drosophila* heart rate and induced arrhythmia (unpublished observations); similar observations of dysrhythmic heartbeat were made in the KCNQ knock-out flies [28].

Our experiments with high concentrations of isradipine, a selective L-type Ca^2+^ channel blocker and an anti- hypertensive medicine, strongly suggest that inhibition of L-type Ca^2+^ channels is a major determinant of SC-791 action on heartbeat. However, what could be the mechanism behind the gradual weakening of contractions?

Reduction in the L-type current is more than just a decrease in a depolarizing current, because Ca^2+^ entering the cell during the action potential is essential not only for maintaining depolarization, but also for excitation-contraction coupling [36] and replenishment of Ca^2+^ stores in the sarcoplasmic reticulum (SR). In the presence of a L-type Ca^2+^ channel blocker, reduced influx of Ca^2+^ through these channels will lower the overall Ca^2+^ load in the lumen during the action potential, which can itself compromise the contraction directly or via reduced Ca^2+^ induced Ca^2+^ release. Intracellular Ca^2+^ is subsequently removed back into the SR by SERCA, and ejected from the cell by the sodium-calcium exchanger or the plasma membrane calcium ATPase. As the share of luminal Ca^2+^ coming from the outside would be lower than in control, the Ca^2+^ removal mechanisms might disproportionally wash out Ca^2+^ coming from the SR, thus gradually depleting the sarcoplasmic Ca^2+^ store and impairing contractions. Interestingly, the notion that coxibs can interfere with muscle contractions by blocking L-type Ca^2+^ channels is supported by experiments with celecoxib in a different experimental system, rat aortic smooth muscle A7r5 cells, where celecoxib inhibits L-type Ca^2+^ channels while augmenting the repolarizing K_V_7.5 current. There, inhibition of L-type Ca^2+^ channels eliminates the arginine vasopressin (AVP)-induced Ca^2+^ spiking and causes concentration-dependent dilation of mesenteric arteries [4].

While inhibition of L-type Ca^2+^ channels by SC-791 could explain most of the observed changes in heartbeat, it could not explain the slowing of contraction velocity. Our experiments with ryanodine suggest that prolongation of contractions in the presence of SC-791 might result from the altered EC coupling, that is, mainly, but not exclusively, from SC-791's interference with the RyR Ca^2+^ release pathway. Application of a saturating concentration of ryanodine (100 μM) could eliminate the rapid component of Ca^2+^ release from intracellular stores, dramatically slowing the contraction velocity and reducing the heart rate. When SC-791 was supplemented to the bath already containing ryanodine, the resultant changes in heartbeat were smaller than expected for independent mechanisms. Although reduction in the Ca^2+^ influx due to inhibition of L-type Ca^2+^ channels by SC-791 could in principle interfere with the RyR-mediated Ca^2+^ induced Ca^2+^ release and slow down the contractions, experiments with isradipine ruled out this possibility. Therefore, it is possible that SC-791 interacted with RyR directly or through one of its regulatory pathways [37]. Importantly, when SC-791 was applied together with ryanodine after a prolonged exposure to ryanodine, the velocity of contractions was further reduced (Fig. 5), suggesting that SC-791 could also target the remaining slow Ca^2+^ release mechanism(s). As a side note, because the effects of ryanodine were also irreversible, such interaction might help explain the irreversibility of SC-791 action on heartbeat (Fig. 3, 5).

In conclusion, this study demonstrates how relatively small differences in molecular action on ion channels of two similar compounds, celecoxib and SC-791, can translate into drastic dissimilarities of drug effects at the organ level, and illustrates the utility of *Drosophila* as a suitable experimental system for basic research on effects of coxibs resulting from inhibition of ion channels.

## Materials and Methods

### Heart beat recording

Strains of *Drosophila melanogaster* were maintained at 21°C. *CS* was used as the wild-type strain. *Shab* denotes the *Shab^3^*, *Drosophila* K_v_2 null-mutant strain [38]. Larval heart beat was monitored at 21°C using a previously described preparation and video recording procedure [8]. In brief, heart rate (HR) was allowed to stabilize for about 5 minutes after the end of dissection. During experiment, HR was counted for 5 min before drug application, for 20 min in the presence of the drug, and for 10 min after washing out the drug. Preparations demonstrating any significant heartbeat irregularity prior to drug application were discarded. Video recordings of heartbeat were made at a resolution of 640×480 pixels at 30 frames/s and data were analyzed as described previously [8]. In brief, a single portion of a video recording (usually 2–20 pixels wide) with clear and unobscured heart wall movement was selected from each video recording for further analysis. This area of interest was transformed into a series of images frame-by-frame using VirtualDub 1.6.5 video editor software by Avery Lee. Location of the wall in each frame was then determined using custom-made software. Resulting waveforms were used to obtain amplitudes and beat-to-beat intervals. This provided a sufficient number (60–300) of events for statistical analysis, which was performed using Microsoft Excel and IgorPro data analysis packages. Coefficient of variation was used as a measure of heartbeat regularity [8].

### Electrophysiology

Voltage-activated K^+^ currents and L-type Ca^2+^ channel currents were recorded from larval body-wall muscles (fiber 12) using two-microelectrode voltage-clamp technique as described previously [24], [39]. The voltage electrode was filled with 2.5 M KCl and the current electrode with a 3∶1 mixture of KCl and potassium citrate. Recording solution for K^+^ currents contained (in mM): NaCl (77.5), KCl (5), MgCl_2_ (4), NaHCO_3_ (2.5), trehalose (5), sucrose (115), and HEPES (5), pH 7.1 [24]. Recording solution to record L-type currents was in addition supplemented with 10 mM BaCl_2_, 20 mM TEA, 1 mM 4-AP, and 100 µM quinidine. SC-791 was purchased from Calbiochem (EMD Chemicals, Inc., 346 NJ) and all other chemicals from Sigma-Aldrich Co. (St. Louis, Missouri USA). Celecoxib was purified as described previously [10]. Because of relatively rapid rundown of ionic currents, each cell was stimulated by an experimental protocol only once.

All experiments were performed at 21°C on a temperature-controlled headstage using a circulating water bath from Forma Scientific. In figures, error bars indicate ± s.e.m. Statistical analysis was performed using a single factor ANOVA test. All values are means ± SEM; (*): *P*<0.05, (**): *P*<0.01.
